# The genome sequence of the Four-dotted Footman, *Cybosia mesomella* (Linnaeus, 1758)

**DOI:** 10.12688/wellcomeopenres.18745.1

**Published:** 2023-01-12

**Authors:** Gavin R. Broad, Natural History Museum Genome Acquisition Lab, Darwin Tree of Life Barcoding collective, Wellcome Sanger Institute Tree of Life programme, Wellcome Sanger Institute Scientific Operations: DNA Pipelines collective, Tree of Life Core Informatics collective, Darwin Tree of Life Consortium

**Affiliations:** 1 Department of Life Sciences, Natural History Museum, London, UK

**Keywords:** Cybosia mesomella, Four-dotted Footman, genome sequence, chromosomal, Lepidoptera

## Abstract

We present a genome assembly from an individual male *Cybosia mesomella* (the Four-dotted Footman; Arthropoda; Insecta; Lepidoptera; Erebidae). The genome sequence is 948 megabases in span. Most of the assembly is scaffolded into 31 chromosomal pseudomolecules, with the Z sex chromosome assembled. The mitochondrial genome has also been assembled and is 15.4 kilobases in length.

## Species taxonomy

Eukaryota; Metazoa; Ecdysozoa; Arthropoda; Hexapoda; Insecta; Pterygota; Neoptera; Endopterygota; Lepidoptera; Glossata; Ditrysia; Noctuoidea; Erebidae; Arctiinae; *Cybosia*; *Cybosia mesomella* (Linnaeus, 1758) (NCBI:txid987918).

## Background

*Cybosia mesomella*, Four-dotted Footman, is widespread but not particularly common in England and Wales, much more local further North. The only species of the genus *Cybosia*, it is found across much of the Palaearctic, but not in the far north or in the far south-west. It is not established in Ireland. 

The English name references the very distinctive feature of two black dots near the outer and trailing edges of each forewing. In describing *Tinea mesomella*, ( Linnaeus, 1758) emphasised the black centre of the underside of the forewing ( Emmet, 1991). Adult moths are most frequently silvery-white with a yellow edge to the forewing, but some are entirely yellow, in Britain most frequently those in the south-east ( Waring *et al.*, 2017). The sequenced individual was of the yellow form. Four-dotted Footman flies from June to early August, with larvae over-wintering. They feed on lichen and algae on stems of woody plants in a variety of habitats, from gardens to woodlands and heath.

While many of the Footmen have been dramatically increasing in their British range and frequency, as a response to improved air quality, *C. mesomella* seems to be increasing only slightly ( Pescott *et al.*, 2015). As the Four-dotted Footman is the only species in the genus, this genome will be particularly useful for comparative genomics of Erebidae, and Lepidoptera more widely. 

## Genome sequence report

**Figure 1.**
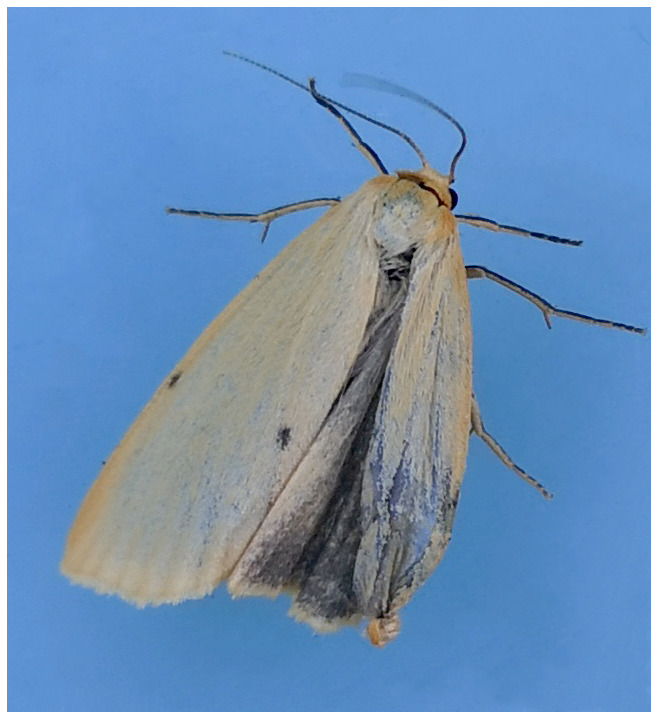
Image of the *Cybosia mesomella* (ilCybMeso1) specimen used for genome sequencing.

The genome was sequenced from one male *Cybosia mesomella* ( Figure 1) collected from a garden in Tonbridge, Kent, UK (latitude 51.19, longitude 0.29). A total of 20-fold coverage in Pacific Biosciences single-molecule HiFi long reads was generated. Primary assembly contigs were scaffolded with chromosome conformation Hi-C data. Manual assembly curation corrected 43 missing joins or mis-joins and removed 10 haplotypic duplications, reducing the assembly length by 0.45% and the scaffold number by 22.73%.

**Figure 2.**
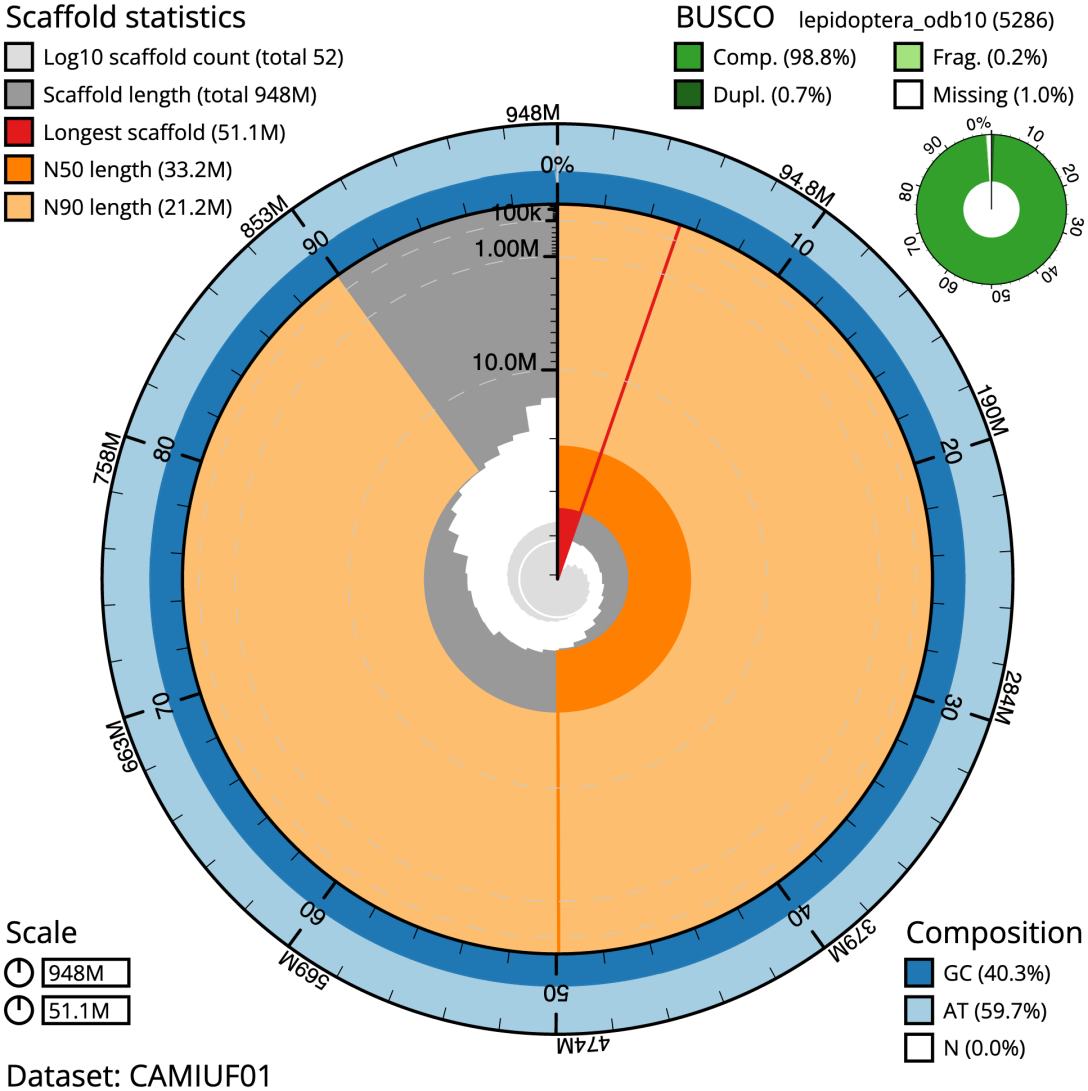
Genome assembly of *Cybosia mesomella*, ilCybMeso1.1: metrics. The BlobToolKit Snailplot shows N50 metrics and BUSCO gene completeness. The main plot is divided into 1,000 size-ordered bins around the circumference with each bin representing 0.1% of the 947,582,557 bp assembly. The distribution of scaffold lengths is shown in dark grey with the plot radius scaled to the longest scaffold present in the assembly (51,108,205 bp, shown in red). Orange and pale-orange arcs show the N50 and N90 sequence lengths (33,223,415 and 21,184,085 bp), respectively. The pale grey spiral shows the cumulative sequence count on a log scale with white scale lines showing successive orders of magnitude. The blue and pale-blue area around the outside of the plot shows the distribution of GC, AT and N percentages in the same bins as the inner plot. A summary of complete, fragmented, duplicated and missing BUSCO genes in the lepidoptera_odb10 set is shown in the top right. An interactive version of this figure is available at. https://blobtoolkit.genomehubs.org/view/ilCybMeso1.1/dataset/CAMIUF01/snail.

The final assembly has a total length of 947.6 Mb in 51 sequence scaffolds with a scaffold N50 of 33.2 Mb ( Table 1). Most (99.92%) of the assembly sequence was assigned to 31 chromosomal-level scaffolds, representing 30 autosomes and the Z sex chromosome ( Figure 2– Figure 5; Table 2). Chromosome-scale scaffolds confirmed by the Hi-C data are named in order of size. The assembly has a BUSCO v5.3.2 ( Manni *et al.*, 2021) completeness of 98.8% (single 98.2%, duplicated 0.7%) using the OrthoDB v10 lepidoptera reference set ( *n* = 5,286). While not fully phased, the assembly deposited is of one haplotype. Contigs corresponding to the second haplotype have also been deposited.

**Table 1.** Genome data for *Cybosia mesomella*, ilCybMeso1.1.

**Project accession data**		
Assembly identifier	ilCybMeso1.1.	
Species	*Cybosia mesomella*	
Specimen	ilCybMeso1	
NCBI taxonomy ID	987918	
BioProject	PRJEB54057	
BioSample ID	SAMEA11025018	
Isolate information		
**Assembly metrics** *		*Benchmark*
Consensus quality (QV)	61.3	*≥ 50*
*k*-mer completeness	100%	*≥ 95%*
BUSCO **	C:98.8%[S:98.2%,D:0.7%], F:0.2%,M:1.0%,n:5,286	*C ≥ 95%*
Percentage of assembly mapped to chromosomes	99.92%	*≥ 95%*
Sex chromosomes	Z	*localised homologous pairs*
Organelles	mitochondrial genome assembled	*complete single alleles*
**Raw data accessions**		
PacificBiosciences SEQUEL II	ERR9924615	
Hi-C Illumina	ERR9930690	
**Genome assembly**		
Assembly accession	GCA_946251805.1	
*Accession of alternate haplotype*	GCA_946251875.1	
Span (Mb)	947.6	
Number of contigs	210	
Contig N50 length (Mb)	9.1	
Number of scaffolds	51	
Scaffold N50 length (Mb)	33.2	
Longest scaffold (Mb)	51.1 (Z chromosome)	

* Assembly metric benchmarks are adapted from column VGP-2020 of “Table 1: Proposed standards and metrics for defining genome assembly quality” from ( Rhie *et al.*, 2021).

** BUSCO scores based on the lepidoptera_odb10 BUSCO set using v5.3.2. C = complete [S = single copy, D = duplicated], F = fragmented, M = missing, n = number of orthologues in comparison. A full set of BUSCO scores is available at https://blobtoolkit.genomehubs.org/view/ilCybMeso1.1/dataset/CAMIUF01/busco.

**Figure 3.**
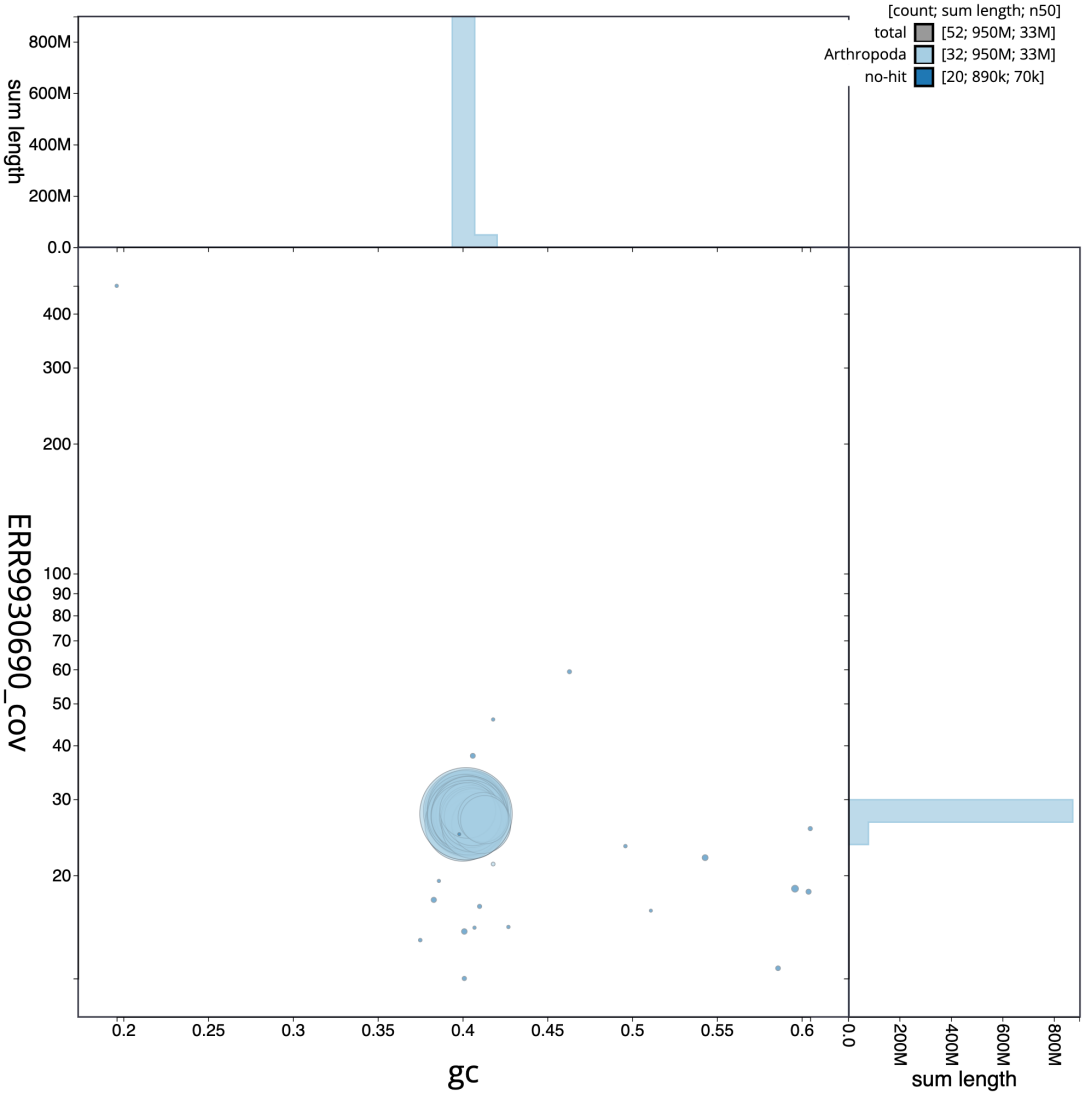
Genome assembly of *Cybosia mesomella*, ilCybMeso1.1: GC coverage. BlobToolKit GC-coverage plot. Scaffolds are coloured by phylum. Circles are sized in proportion to scaffold length. Histograms show the distribution of scaffold length sum along each axis. An interactive version of this figure is available at https://blobtoolkit.genomehubs.org/view/ilCybMeso1.1/dataset/CAMIUF01/blob.

**Figure 4.**
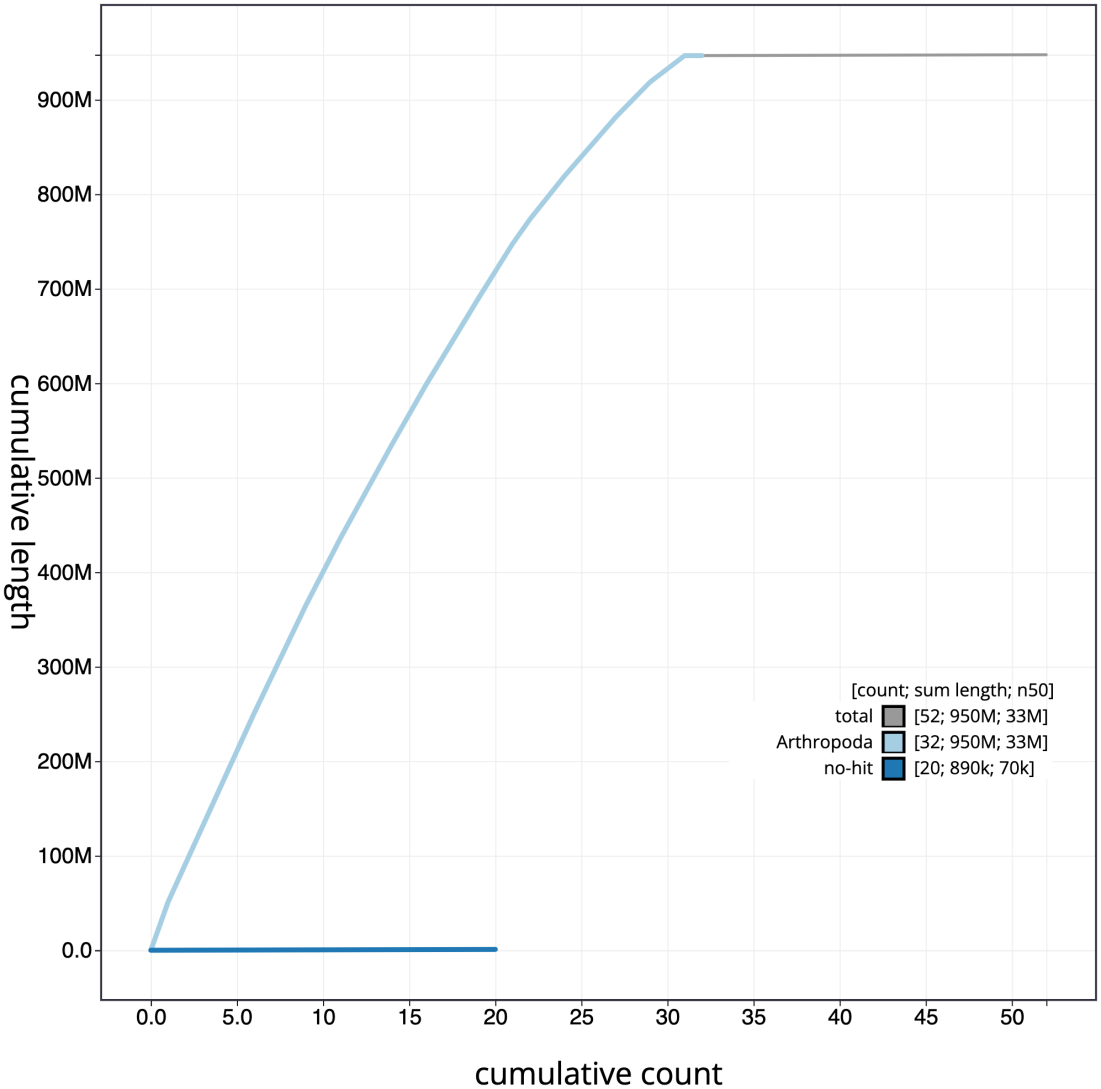
Genome assembly of *Cybosia mesomella*, ilCybMeso1.1: cumulative sequence. BlobToolKit cumulative sequence plot. The grey line shows cumulative length for all scaffolds. Coloured lines show cumulative lengths of scaffolds assigned to each phylum using the buscogenes taxrule. An interactive version of this figure is available at https://blobtoolkit.genomehubs.org/view/ilCybMeso1.1/dataset/CAMIUF01/cumulative.

**Figure 5.**
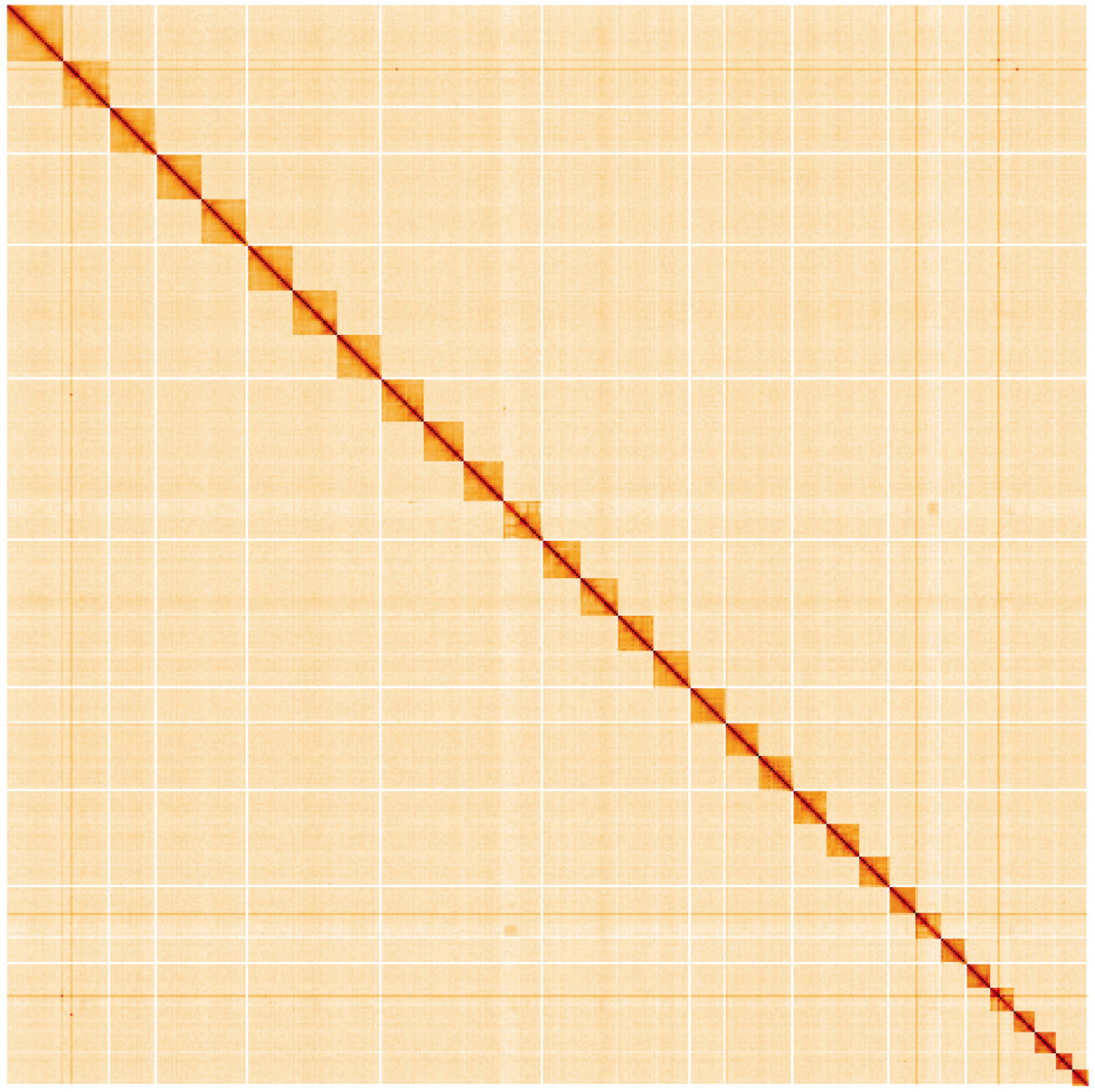
Genome assembly of *Cybosia mesomella*, ilCybMeso1.1: Hi-C contact map. Hi-C contact map of the ilCybMeso1.1 assembly, visualised using HiGlass. Chromosomes are shown in order of size from left to right and top to bottom. An interactive version of this figure may be viewed at https://genome-note-higlass.tol.sanger.ac.uk/l/?d=BiXkTo_iQGW7JQyZ1wWBGg.

**Table 2.** Chromosomal pseudomolecules in the genome assembly of *Cybosia mesomella*, ilCybMeso1.

INSDC accession	Chromosome	Size (Mb)	GC%
OX276389.1	1	40.42	40.4
OX276390.1	2	40.07	40.2
OX276391.1	3	40	40.1
OX276392.1	4	39.82	40.3
OX276393.1	5	39.42	40.3
OX276394.1	6	38.84	40.3
OX276395.1	7	38.2	40.3
OX276396.1	8	37.15	40.3
OX276397.1	9	36.08	40.2
OX276398.1	10	34.6	40.4
OX276399.1	11	33.7	40
OX276400.1	12	33.22	40.1
OX276401.1	13	32.49	40.1
OX276402.1	14	31.93	40.1
OX276403.1	15	31.81	40.3
OX276404.1	16	30.33	40.4
OX276405.1	17	30.22	40.4
OX276406.1	18	29.87	40.3
OX276407.1	19	29.26	40.5
OX276408.1	20	28.83	40.2
OX276409.1	21	25.75	40.1
OX276410.1	22	23.5	40.5
OX276411.1	23	22.15	40.6
OX276412.1	24	21.25	40.5
OX276413.1	25	21.18	40.5
OX276414.1	26	20.43	41.1
OX276415.1	27	18.99	40.6
OX276416.1	28	18.19	40.2
OX276417.1	29	14.37	41.2
OX276418.1	30	13.48	41.3
OX276388.1	Z	51.11	40.2
OX276419.1	MT	0.02	19.8
-	unplaced	0.9	49.2

## Methods

### Sample acquisition and nucleic acid extraction

A male *Cybosia mesomella* specimen (ilCybMeso1) was collected from a garden in Tonbridge, Kent, UK (latitude 51.19, longitude 0.29), using an actinic light. The specimen was collected and identified by Gavin Broad (Natural History Museum) and snap-frozen at –80°C.

DNA was extracted at the Tree of Life laboratory, Wellcome Sanger Institute (WSI). The ilCybMeso1 sample was weighed and dissected on dry ice with tissue set aside for Hi-C sequencing. Thorax tissue was disrupted using a Nippi Powermasher fitted with a BioMasher pestle. High molecular weight (HMW) DNA was extracted using the Qiagen MagAttract HMW DNA extraction kit. HMW DNA was sheared into an average fragment size of 12–20 kb in a Megaruptor 3 system with speed setting 30. Sheared DNA was purified by solid-phase reversible immobilisation using AMPure PB beads with a 1.8X ratio of beads to sample to remove the shorter fragments and concentrate the DNA sample. The concentration of the sheared and purified DNA was assessed using a Nanodrop spectrophotometer and Qubit Fluorometer and Qubit dsDNA High Sensitivity Assay kit. Fragment size distribution was evaluated by running the sample on the FemtoPulse system.

### Sequencing

A Pacific Biosciences HiFi circular consensus DNA sequencing library was constructed according to the manufacturers’ instructions. DNA sequencing was performed by the Scientific Operations core at the WSI on a Pacific Biosciences SEQUEL II (HiFi) instrument. Hi-C data were also generated from head tissue of ilCybMeso1using the Arima v2 kit and sequenced on the Illumina NovaSeq 6000 instrument.

### Genome assembly

Assembly was carried out with Hifiasm ( Cheng *et al.*, 2021) and haplotypic duplication was identified and removed with purge_dups ( Guan *et al.*, 2020). The assembly was then scaffolded with Hi-C data ( Rao *et al.*, 2014) using YaHS ( Zhou *et al.*, 2022). The assembly was checked for contamination as described previously ( Howe *et al.*, 2021). Manual curation was performed using HiGlass ( Kerpedjiev *et al.*, 2018) and Pretext ( Harry, 2022). The mitochondrial genome was assembled using MitoHiFi ( Uliano-Silva *et al.*, 2021), which performed annotation using MitoFinder ( Allio *et al.*, 2020). The genome was analysed and BUSCO scores generated within the BlobToolKit environment ( Challis *et al.*, 2020). Table 3 contains a list of all software tool versions used, where appropriate.

**Table 3.** Software tools and versions used.

Software tool	Version	Source
BlobToolKit	3.4.0	Challis *et al.*, 2020
Hifiasm	0.16.1-r375	Cheng *et al.*, 2021
HiGlass	1.11.6	Kerpedjiev *et al.*, 2018
MitoHiFi	2	Uliano-Silva *et al.*, 2021
PretextView	0.2	Harry, 2022
purge_dups	1.2.3	Guan *et al.*, 2020
YaHS	yahs-1.1.91eebc2	Zhou *et al.*, 2022

### Ethics/compliance issues

The materials that have contributed to this genome note have been supplied by a Darwin Tree of Life Partner. The submission of materials by a Darwin Tree of Life Partner is subject to the Darwin Tree of Life Project Sampling Code of Practice. By agreeing with and signing up to the Sampling Code of Practice, the Darwin Tree of Life Partner agrees they will meet the legal and ethical requirements and standards set out within this document in respect of all samples acquired for, and supplied to, the Darwin Tree of Life Project. Each transfer of samples is further undertaken according to a Research Collaboration Agreement or Material Transfer Agreement entered into by the Darwin Tree of Life Partner, Genome Research Limited (operating as the Wellcome Sanger Institute), and in some circumstances other Darwin Tree of Life collaborators.

## Data Availability

European Nucleotide Archive: *Cybosia mesomella*. Accession number PRJEB54057; https://identifiers.org/ena.embl/PRJEB54057 ( Wellcome Sanger Institute, 2022). The genome sequence is released openly for reuse. The *Cybosia mesomella* genome sequencing initiative is part of the Darwin Tree of Life (DToL) project. All raw sequence data and the assembly have been deposited in INSDC databases. The genome will be annotated using available RNA-Seq data and presented through the Ensembl pipeline at the European Bioinformatics Institute. Raw data and assembly accession identifiers are reported in Table 1.

## Author information

Members of the Natural History Museum Genome Acquisition Lab are listed here: https://doi.org/10.5281/zenodo.4790042.

Members of the Darwin Tree of Life Barcoding collective are listed here: https://doi.org/10.5281/zenodo.4893703.

Members of the Wellcome Sanger Institute Tree of Life programme are listed here: https://doi.org/10.5281/zenodo.4783585.

Members of Wellcome Sanger Institute Scientific Operations: DNA Pipelines collective are listed here: https://doi.org/10.5281/zenodo.4790455.

Members of the Tree of Life Core Informatics collective are listed here: https://doi.org/10.5281/zenodo.5013541.

Members of the Darwin Tree of Life Consortium are listed here: https://doi.org/10.5281/zenodo.4783558.
