# Supplementary material for: The genome sequence of the Four-dotted Footman, Cybosia mesomella (Linnaeus, 1758)
Source: Wellcome Open Res. 2023 Jan 12;8:18. [Version 1] doi: 10.12688/wellcomeopenres.18745.1 (PMC10280026; doi:10.12688/wellcomeopenres.18745.1)
Supplement: Peer review history

# Peer review: Reviewer response for version 1

**DOI:** 10.21956/wellcomeopenres.20784.r58145

**Published:** 2023-06-13

**Authors:** Nicolas J. Dowdy, Kathryn Sullivan

**Affiliations:** 1 Department of Biology, Wake Forest University, Winston-Salem, NC, USA; 2 Biological Sciences, Marquette University, Milwaukee, Wisconsin, USA; 3 Zoology, Milwaukee Public Museum, Milwaukee, Wisconsin, USA

This is a fantastic addition to the genomic resources of the Lepidoptera of Britain and Ireland. I have just a few notes:

 Taxonomy: additionally this species is in the tribe Lithosiini, and the subtribe Lithosiina.

 There are currently 2 species in the genus Cybosia. Lithosia gynaegrapha de Joannis, 1930 was provisionally moved to this genus based on genitalic and morphological similarities by Dubatolov et al., 2016 ^1^.

 pg. 3 Methods:

 Is/was specimen deposited? Is there a voucher remaining? More info on that in methods than genome sequence report.

 pg. 4 Table 1 Isolate information is blank--BioSampleID indicates this was the whole organism but text only reveals thorax tissue used

Are sufficient details of methods and materials provided to allow replication by others?

Yes

Is the rationale for creating the dataset(s) clearly described?

Yes

Are the datasets clearly presented in a useable and accessible format?

Yes

Are the protocols appropriate and is the work technically sound?

Yes

Reviewer Expertise:

Phylogenomics, Systematics, Taxonomy, Behavioral & Chemical Ecology

We confirm that we have read this submission and believe that we have an appropriate level of expertise to confirm that it is of an acceptable scientific standard.

## References

[1] : Revision of Lithosia Fabricius, 1798 and Conilepia Hampson, 1900 (Lepidoptera, Arctiidae). *Zootaxa* .2016;4107(2) : 10.11646/zootaxa.4107.2.3 175-96 10.11646/zootaxa.4107.2.3 27394813

# Peer review: Reviewer response for version 1

**DOI:** 10.21956/wellcomeopenres.20784.r58146

**Published:** 2023-06-13

**Authors:** Sivasankaran Kuppusamy

**Affiliations:** 1 Loyola Collège, Chennai, India

**Comments on the Manuscript**

 I would like to appreciate the authors for compiling the Four-dotted Footman, *Cybosia mesomella* complete genome sequence. The authors have employed the proper software, genome assembly techniques, and whole genome sequencing methods.

 The species taxonomy can be given as Eukaryota; Metazoa; Ecdysozoa, Arthropoda; Insecta; Pterygota; Neoptera; Endopterygota; Lepidoptera; Glossata; Ditrysia; Obtectomera; Macroheterocera; Noctuoidea; Erebidae; Arctiinae; *Cyboscia mesomella.*

 In the “Background” of the manuscript, the first line of the second paragraph can be modified as, “The species name reference is very distinctive feature of two prominent black dots subcostal and anal region of forewing”.

 Following, The author has mistakenly mentioned the genus name *Tinea* instead of *Cybosia* in the second line *.* The genus name must be changed.

 The fifth sentence can be changed as “The four-dotted Footman moths are flying from…………"

 Genome sequence report:

 The first sentence can be changed “The whole genome was sequenced from an adult male specimen of *Cybosia mesomella* …"

 Methods:

 Second paragraph of first line can be start “The genomic DNA was extracted at the…."

 The sequence length and number of scaffolds were properly mentioned in the legends. 

 The manuscript is well written overall, and it can be accepted for indexing.

Are sufficient details of methods and materials provided to allow replication by others?

Yes

Is the rationale for creating the dataset(s) clearly described?

Yes

Are the datasets clearly presented in a useable and accessible format?

Yes

Are the protocols appropriate and is the work technically sound?

Yes

Reviewer Expertise:

Taxonomy and Phylogenetic analysis of superfamily Noctuoidea moths using mitochondrial genome sequences.

I confirm that I have read this submission and believe that I have an appropriate level of expertise to confirm that it is of an acceptable scientific standard.
